# Supplementary material for: The possible molecular mechanism underlying the involvement of the variable shear factor QKI in the epithelial-mesenchymal transformation of oesophageal cancer
Source: PLoS One. 2023 Jul 10;18(7):e0288403. doi: 10.1371/journal.pone.0288403 (PMC10332600; doi:10.1371/journal.pone.0288403)
Supplement: S1 File — (ZIP) [file pone.0288403.s001.zip › supporting information/TCGA-ESCA/GSEA/gsea_report_for_highExp_1669339701317.html]

Report for highExp 1669339701317 [GSEA]

| GS  follow link to MSigDB | GS DETAILS | SIZE | ES | NES | NOM p-val | FDR q-val | FWER p-val | RANK AT MAX | LEADING EDGE || 1 | HALLMARK\_EPITHELIAL\_MESENCHYMAL\_TRANSITION | Details ... | 200 | 0.66 | 3.31 | 0.000 | 0.000 | 0.000 | 3379 | tags=69%, list=21%, signal=85% |
| 2 | HALLMARK\_INFLAMMATORY\_RESPONSE | Details ... | 197 | 0.45 | 2.25 | 0.000 | 0.000 | 0.000 | 4037 | tags=46%, list=25%, signal=60% |
| 3 | HALLMARK\_TNFA\_SIGNALING\_VIA\_NFKB | Details ... | 199 | 0.44 | 2.23 | 0.000 | 0.000 | 0.000 | 3695 | tags=43%, list=23%, signal=55% |
| 4 | HALLMARK\_HYPOXIA | Details ... | 193 | 0.42 | 2.10 | 0.000 | 0.000 | 0.000 | 3084 | tags=36%, list=19%, signal=44% |
| 5 | HALLMARK\_APICAL\_JUNCTION | Details ... | 193 | 0.41 | 2.06 | 0.000 | 0.000 | 0.000 | 3286 | tags=40%, list=20%, signal=50% |
| 6 | HALLMARK\_ALLOGRAFT\_REJECTION | Details ... | 193 | 0.40 | 2.00 | 0.000 | 0.000 | 0.000 | 5056 | tags=53%, list=31%, signal=76% |
| 7 | HALLMARK\_INTERFERON\_GAMMA\_RESPONSE | Details ... | 199 | 0.38 | 1.93 | 0.000 | 0.000 | 0.002 | 4740 | tags=48%, list=29%, signal=66% |
| 8 | HALLMARK\_ANGIOGENESIS | Details ... | 35 | 0.50 | 1.89 | 0.000 | 0.001 | 0.004 | 3406 | tags=43%, list=21%, signal=54% |
| 9 | HALLMARK\_HEDGEHOG\_SIGNALING | Details ... | 35 | 0.51 | 1.87 | 0.000 | 0.001 | 0.005 | 2442 | tags=40%, list=15%, signal=47% |
| 10 | HALLMARK\_MYOGENESIS | Details ... | 178 | 0.38 | 1.85 | 0.000 | 0.001 | 0.005 | 4655 | tags=50%, list=28%, signal=69% |
| 11 | HALLMARK\_UV\_RESPONSE\_DN | Details ... | 144 | 0.37 | 1.78 | 0.000 | 0.001 | 0.011 | 3296 | tags=40%, list=20%, signal=49% |
| 12 | HALLMARK\_IL6\_JAK\_STAT3\_SIGNALING | Details ... | 85 | 0.39 | 1.69 | 0.000 | 0.004 | 0.035 | 5040 | tags=51%, list=31%, signal=73% |
| 13 | HALLMARK\_IL2\_STAT5\_SIGNALING | Details ... | 196 | 0.32 | 1.58 | 0.002 | 0.011 | 0.097 | 3300 | tags=32%, list=20%, signal=40% |
| 14 | HALLMARK\_WNT\_BETA\_CATENIN\_SIGNALING | Details ... | 41 | 0.40 | 1.53 | 0.032 | 0.016 | 0.152 | 2904 | tags=34%, list=18%, signal=41% |
| 15 | HALLMARK\_TGF\_BETA\_SIGNALING | Details ... | 53 | 0.37 | 1.47 | 0.023 | 0.027 | 0.253 | 2180 | tags=26%, list=13%, signal=30% |
| 16 | HALLMARK\_G2M\_CHECKPOINT | Details ... | 199 | 0.28 | 1.42 | 0.009 | 0.041 | 0.379 | 5932 | tags=42%, list=36%, signal=65% |
| 17 | HALLMARK\_COMPLEMENT | Details ... | 196 | 0.26 | 1.31 | 0.023 | 0.093 | 0.695 | 4094 | tags=38%, list=25%, signal=50% |
| 18 | HALLMARK\_COAGULATION | Details ... | 124 | 0.28 | 1.31 | 0.049 | 0.090 | 0.701 | 3029 | tags=30%, list=19%, signal=36% |
| 19 | HALLMARK\_KRAS\_SIGNALING\_UP | Details ... | 196 | 0.26 | 1.30 | 0.048 | 0.090 | 0.721 | 3333 | tags=30%, list=20%, signal=37% |
| 20 | HALLMARK\_MITOTIC\_SPINDLE | Details ... | 198 | 0.26 | 1.29 | 0.033 | 0.095 | 0.760 | 3494 | tags=29%, list=21%, signal=37% |
| 21 | HALLMARK\_E2F\_TARGETS |  | 200 | 0.26 | 1.29 | 0.031 | 0.090 | 0.760 | 6581 | tags=49%, list=40%, signal=80% |
| 22 | HALLMARK\_APOPTOSIS |  | 159 | 0.26 | 1.28 | 0.051 | 0.095 | 0.802 | 4881 | tags=41%, list=30%, signal=58% |
| 23 | HALLMARK\_P53\_PATHWAY |  | 198 | 0.24 | 1.23 | 0.053 | 0.137 | 0.901 | 4686 | tags=35%, list=29%, signal=49% |
| 24 | HALLMARK\_APICAL\_SURFACE |  | 43 | 0.32 | 1.22 | 0.180 | 0.135 | 0.909 | 4216 | tags=37%, list=26%, signal=50% |
| 25 | HALLMARK\_INTERFERON\_ALPHA\_RESPONSE |  | 97 | 0.26 | 1.17 | 0.178 | 0.195 | 0.976 | 5258 | tags=42%, list=32%, signal=62% |
| 26 | HALLMARK\_MTORC1\_SIGNALING |  | 199 | 0.21 | 1.07 | 0.272 | 0.375 | 1.000 | 5368 | tags=41%, list=33%, signal=60% |
| 27 | HALLMARK\_NOTCH\_SIGNALING |  | 31 | 0.30 | 1.06 | 0.344 | 0.374 | 1.000 | 3046 | tags=45%, list=19%, signal=55% |
| 28 | HALLMARK\_HEME\_METABOLISM |  | 182 | 0.18 | 0.89 | 0.745 | 0.838 | 1.000 | 3148 | tags=22%, list=19%, signal=27% |
| 29 | HALLMARK\_REACTIVE\_OXYGEN\_SPECIES\_PATHWAY |  | 48 | 0.21 | 0.82 | 0.781 | 0.970 | 1.000 | 4917 | tags=35%, list=30%, signal=50% |
| 30 | HALLMARK\_PI3K\_AKT\_MTOR\_SIGNALING |  | 101 | 0.18 | 0.80 | 0.855 | 0.964 | 1.000 | 3265 | tags=24%, list=20%, signal=30% |
| 31 | HALLMARK\_KRAS\_SIGNALING\_DN |  | 170 | 0.16 | 0.79 | 0.945 | 0.950 | 1.000 | 2614 | tags=18%, list=16%, signal=21% |
| 32 | HALLMARK\_MYC\_TARGETS\_V1 |  | 199 | 0.11 | 0.55 | 1.000 | 0.999 | 1.000 | 6469 | tags=39%, list=40%, signal=64% |
Table: Gene sets enriched in phenotype **highExp (82 samples)**[plain text format]****

  
