# Supplementary material for: The possible molecular mechanism underlying the involvement of the variable shear factor QKI in the epithelial-mesenchymal transformation of oesophageal cancer
Source: PLoS One. 2023 Jul 10;18(7):e0288403. doi: 10.1371/journal.pone.0288403 (PMC10332600; doi:10.1371/journal.pone.0288403)
Supplement: S1 File — (ZIP) [file pone.0288403.s001.zip › supporting information/TCGA-ESCA/GSEA/gsea_report_for_lowExp_1669339701317.html]

Report for lowExp 1669339701317 [GSEA]

| GS  follow link to MSigDB | GS DETAILS | SIZE | ES | NES | NOM p-val | FDR q-val | FWER p-val | RANK AT MAX | LEADING EDGE || 1 | HALLMARK\_OXIDATIVE\_PHOSPHORYLATION | Details ... | 200 | -0.50 | -2.44 | 0.000 | 0.000 | 0.000 | 3974 | tags=49%, list=24%, signal=64% |
| 2 | HALLMARK\_FATTY\_ACID\_METABOLISM | Details ... | 151 | -0.40 | -1.91 | 0.000 | 0.000 | 0.001 | 2477 | tags=32%, list=15%, signal=37% |
| 3 | HALLMARK\_ADIPOGENESIS | Details ... | 196 | -0.37 | -1.81 | 0.000 | 0.001 | 0.004 | 3394 | tags=37%, list=21%, signal=46% |
| 4 | HALLMARK\_XENOBIOTIC\_METABOLISM | Details ... | 187 | -0.37 | -1.80 | 0.000 | 0.001 | 0.006 | 2280 | tags=29%, list=14%, signal=33% |
| 5 | HALLMARK\_PEROXISOME | Details ... | 103 | -0.39 | -1.73 | 0.000 | 0.003 | 0.020 | 3386 | tags=39%, list=21%, signal=49% |
| 6 | HALLMARK\_BILE\_ACID\_METABOLISM | Details ... | 107 | -0.38 | -1.73 | 0.000 | 0.003 | 0.021 | 3386 | tags=41%, list=21%, signal=52% |
| 7 | HALLMARK\_ESTROGEN\_RESPONSE\_EARLY | Details ... | 199 | -0.33 | -1.61 | 0.002 | 0.007 | 0.068 | 2428 | tags=28%, list=15%, signal=33% |
| 8 | HALLMARK\_GLYCOLYSIS | Details ... | 195 | -0.29 | -1.43 | 0.008 | 0.037 | 0.353 | 2786 | tags=27%, list=17%, signal=32% |
| 9 | HALLMARK\_ESTROGEN\_RESPONSE\_LATE | Details ... | 198 | -0.28 | -1.38 | 0.006 | 0.052 | 0.495 | 2240 | tags=24%, list=14%, signal=28% |
| 10 | HALLMARK\_MYC\_TARGETS\_V2 | Details ... | 56 | -0.33 | -1.33 | 0.079 | 0.078 | 0.689 | 5557 | tags=48%, list=34%, signal=73% |
| 11 | HALLMARK\_CHOLESTEROL\_HOMEOSTASIS | Details ... | 74 | -0.30 | -1.24 | 0.099 | 0.147 | 0.917 | 2095 | tags=22%, list=13%, signal=25% |
| 12 | HALLMARK\_PROTEIN\_SECRETION | Details ... | 95 | -0.27 | -1.20 | 0.134 | 0.192 | 0.970 | 4036 | tags=37%, list=25%, signal=49% |
| 13 | HALLMARK\_PANCREAS\_BETA\_CELLS | Details ... | 35 | -0.32 | -1.16 | 0.229 | 0.243 | 0.993 | 5152 | tags=49%, list=31%, signal=71% |
| 14 | HALLMARK\_UV\_RESPONSE\_UP | Details ... | 156 | -0.24 | -1.14 | 0.198 | 0.262 | 0.995 | 1866 | tags=15%, list=11%, signal=17% |
| 15 | HALLMARK\_DNA\_REPAIR | Details ... | 148 | -0.22 | -1.04 | 0.374 | 0.434 | 1.000 | 4361 | tags=30%, list=27%, signal=40% |
| 16 | HALLMARK\_ANDROGEN\_RESPONSE | Details ... | 97 | -0.21 | -0.94 | 0.615 | 0.677 | 1.000 | 1356 | tags=14%, list=8%, signal=16% |
| 17 | HALLMARK\_UNFOLDED\_PROTEIN\_RESPONSE | Details ... | 113 | -0.20 | -0.89 | 0.716 | 0.774 | 1.000 | 3052 | tags=20%, list=19%, signal=25% |
| 18 | HALLMARK\_SPERMATOGENESIS | Details ... | 107 | -0.16 | -0.73 | 0.957 | 0.965 | 1.000 | 2740 | tags=17%, list=17%, signal=20% |
Table: Gene sets enriched in phenotype **lowExp (81 samples)**[plain text format]****

  
