# Supplementary figures and images for: The possible molecular mechanism underlying the involvement of the variable shear factor QKI in the epithelial-mesenchymal transformation of oesophageal cancer
Source: PLoS One. 2023 Jul 10;18(7):e0288403. doi: 10.1371/journal.pone.0288403 (PMC10332600; doi:10.1371/journal.pone.0288403)

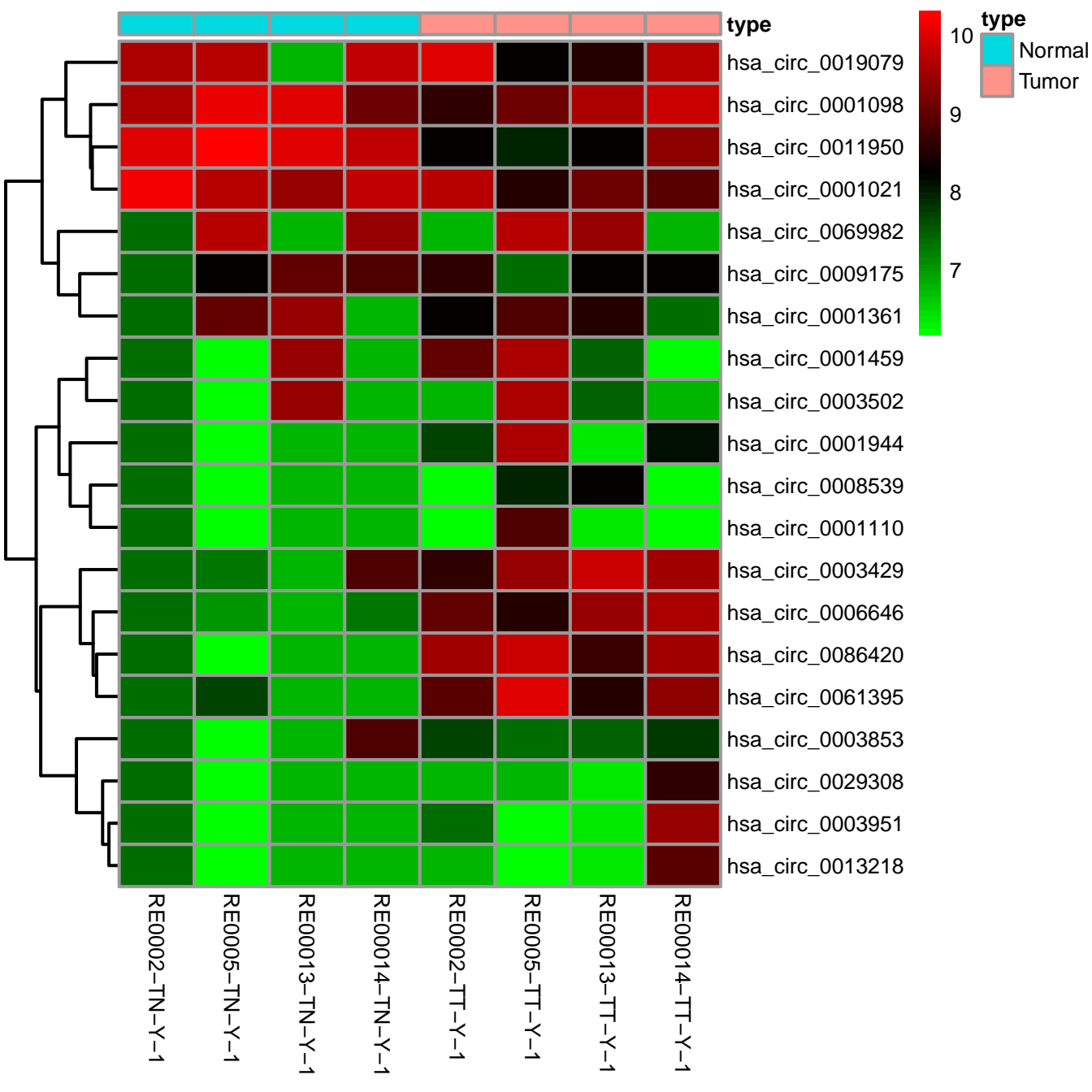

Supplement: S1 File — (ZIP) [file pone.0288403.s001.zip › supporting information/GSE189830/heatmap4.pdf]

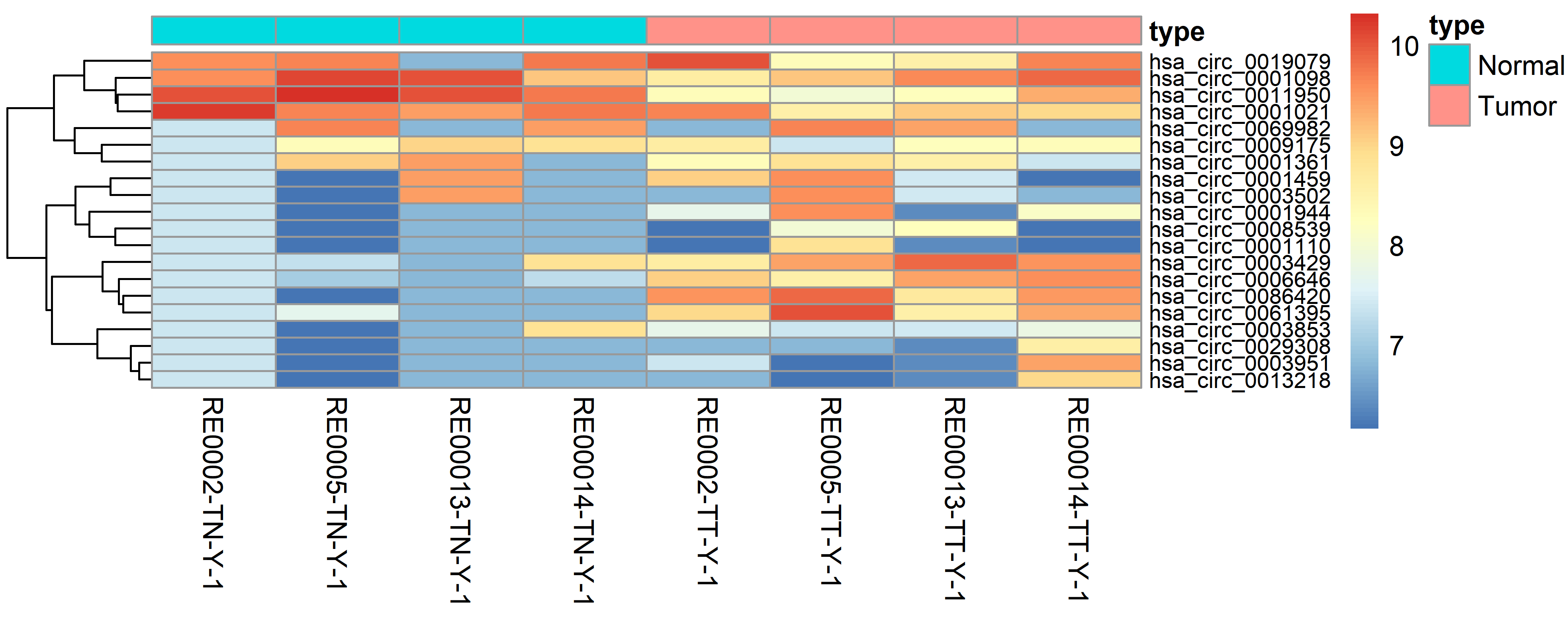

Supplement: S1 File — (ZIP) [file pone.0288403.s001.zip › supporting information/GSE189830/heatmap4.tiff]

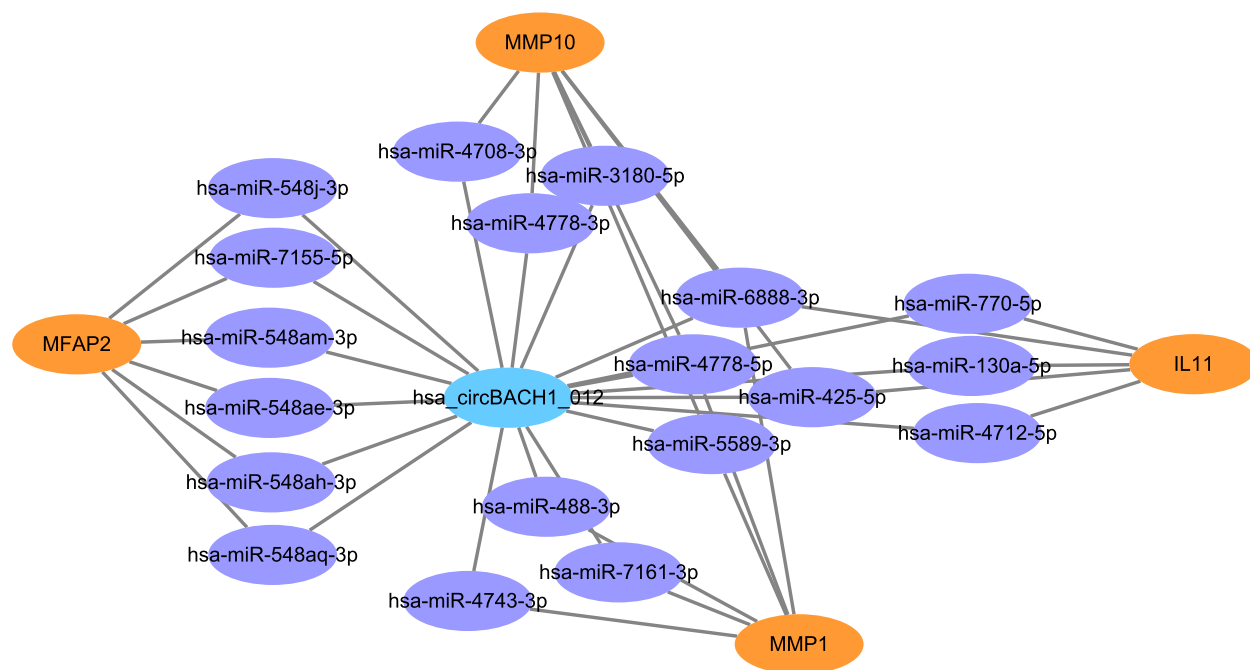

Supplement: S1 File — (ZIP) [file pone.0288403.s001.zip › supporting information/network/circRNA-miRNA-mRNA/hsa_circBACH1_012下游miRNA-miRNA调控网络.pdf]

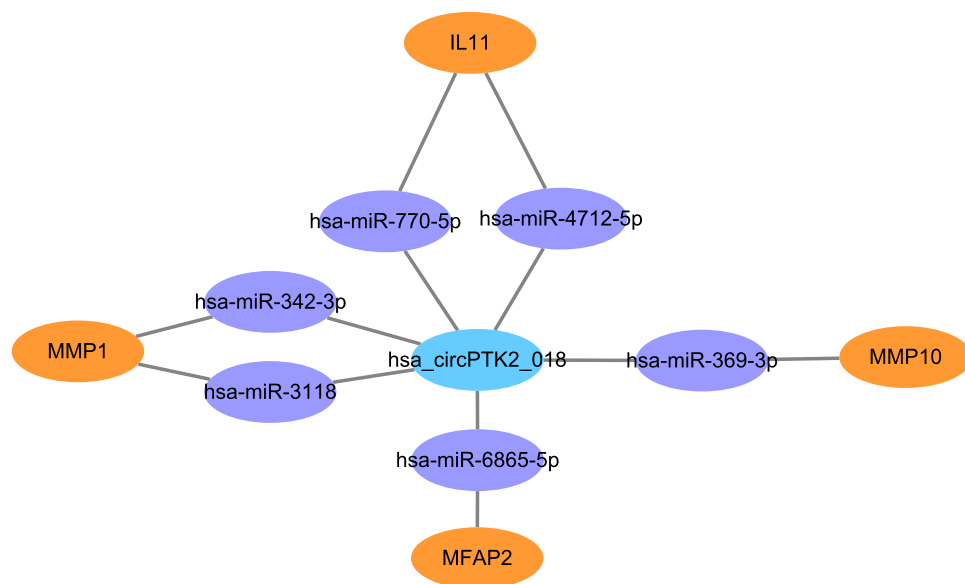

Supplement: S1 File — (ZIP) [file pone.0288403.s001.zip › supporting information/network/circRNA-miRNA-mRNA/hsa_circPTK2_018下游miRNA-miRNA调控网络.pdf]

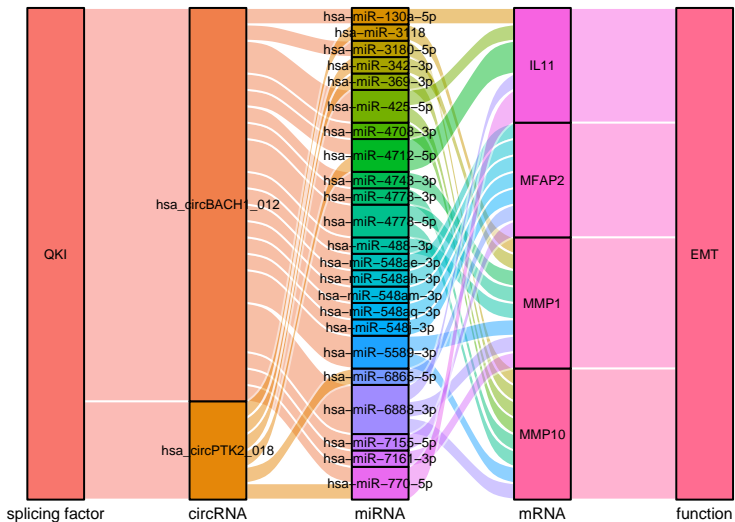

Supplement: S1 File — (ZIP) [file pone.0288403.s001.zip › supporting information/network/Sangjitu Xiantao Academic Online Tool Drawing/桑基图_2022-11-25_17_11_32.pdf]

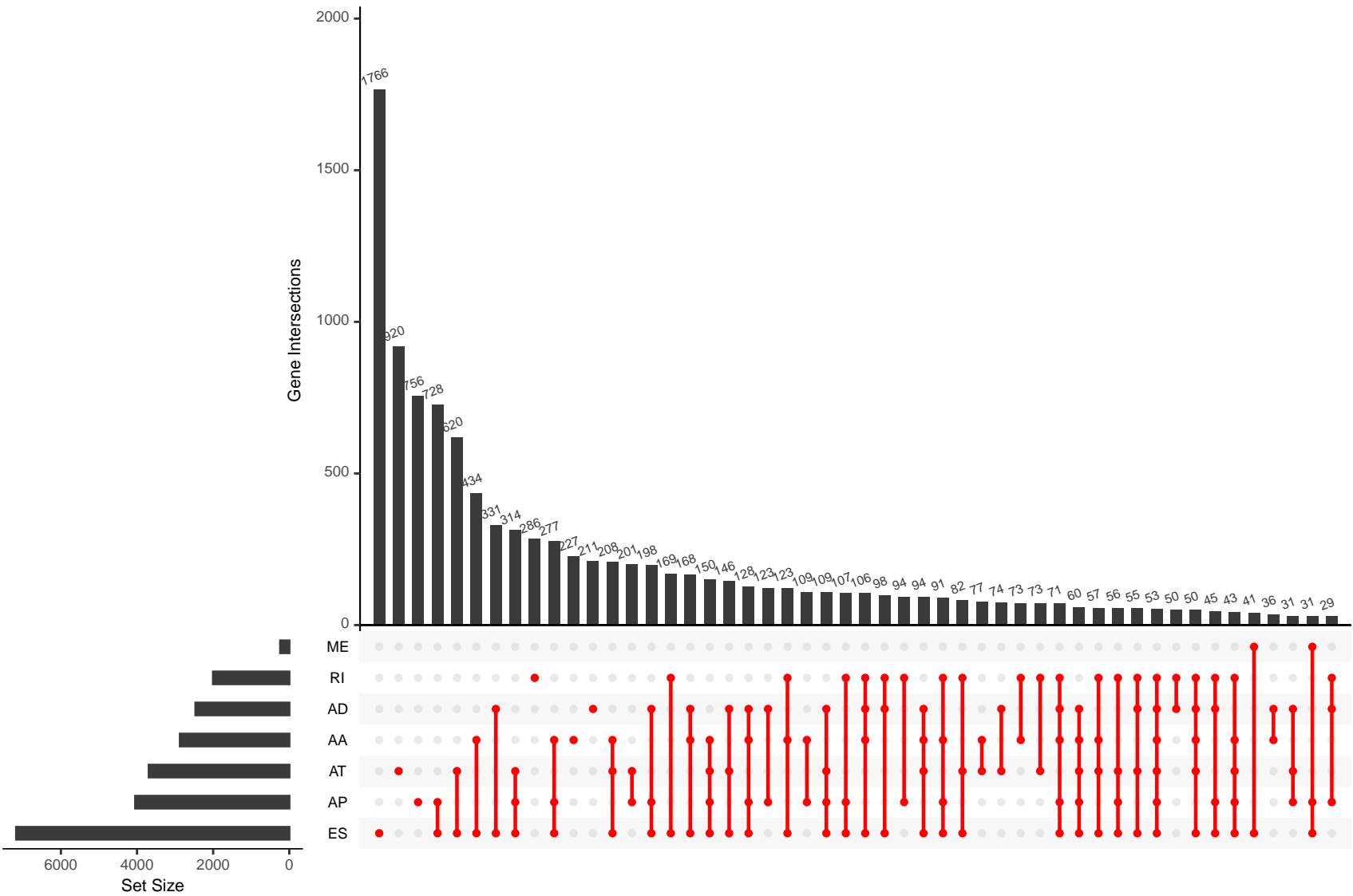

Supplement: S1 File — (ZIP) [file pone.0288403.s001.zip › supporting information/TCGA-ESCA/Variable shear/upset.pdf]
