# Supplementary material for: The possible molecular mechanism underlying the involvement of the variable shear factor QKI in the epithelial-mesenchymal transformation of oesophageal cancer
Source: PLoS One. 2023 Jul 10;18(7):e0288403. doi: 10.1371/journal.pone.0288403 (PMC10332600; doi:10.1371/journal.pone.0288403)
Supplement: Attachment — Submitted filename: Response to Reviewers.docx [file pone.0288403.s002.docx]

a. The abstract conclusion is too short, only one sentence.

**Response:** Thank you for your comments from the academic editor. I have revised the original text and marked it in red. The content is as follows:

Variable shear factor QKI promotes hsa_circ_0006646 and hsa_circ_0061395 generation, and downstream related miRNAs can relieve the targeted inhibition of EMT-related genes (IL11, MFAP2, MMP10, MMP1) and promote the occurrence and development of oesophageal cancer, providing a new theoretical basis for screening prognostic markers of oesophageal cancer patients.
b. “In 2020, there will be 604000 new cases of oesophageal cancer and more than 540000 deaths”, should be 604,000 and 540,000.

**Response:** Thank you for your comments from the academic editor. I have revised the original text and marked it in red.
c. P. 11: briefly describe what FPKM stands for. Also what is ASE?

**Response:** We are sorry for this mistake. In fact, the data format used in the differential analysis part of this study is TPM not FPKM, and we have provided its full names “transcripts per million (TPM) reads”. Meanwhile, we have unified the descriptions between the figure and methods. In addition, we have already detailed ASE “atomic simulation environment” at its first appearance in the text.

d. P 11: “Since these data were obtained from public databases, no ethical approval or informed consent was needed.” This should go into some sort of ethics declaration at the end of the paper.

**Response:** Thank you for your comments from the academic editor. I have added an ethical declaration at the end of the paper and marked it in red. The content is as follows:

Ethics Declaration

Since TCGA、GTEx 、GEO databases were obtained from public databases, no ethical approval or informed consent was needed.

e. P. 11: describe what a shear diagram is briefly.

**Response:** Thank you for your comments from the academic editor. The content is as follows:

We have revised it to “an UpSet diagram of alternative splicing (schematic diagram of different alternative splicing methods) was drawn”.

f. P. 12: how did you combine the TCGA and GTEx datasets (Results, 1st paragraph)?

**Response:** Thank you for your comments from the academic editor. In the section of Screening of differentially expressed mRNAs and circRNAs, we have added details “The ComBat R package was used to remove the batch effect of data collected from TCGA and GTEx databases, after which data were merged.”.

g. “upregulated compared with that in normal control samples” should be compared with those in …

**Response:** Thank you for your comments from the academic editor. I have revised the original text and marked it in red. The content is as follows:

After data correction, an analysis of differential expression showed that the expression of QKI in tumour tissue samples of oesophageal cancer patients was significantly upregulated compared with those in normal control samples.
h. P. 13: Isn’t 0.2 > |r| too low of a threshold?

**Response:** Thank you for your comments from the academic editor. In the correlation analysis results, we observed that the |r| of most results is mainly distributed within 0.1-0.3, that is, weak correlation. It can also be seen from Table 1 that if the screening criterion was set to |r| > 0.3, only 4 coding genes were screened, which was not conducive to intersecting with another gene set. In general, when multiple screening conditions exist at the same time, we appropriately lowered the screening criteria of |r|, and finally determined |r| > 0.2 as the threshold.
i. P. 17: put the targets of has_circ_0006646 into a table, and refer to this table in the text (suggestion).

**Response:** Thank you for your comments from the academic editor. The targets of has_circ_0006646 has been shown in Table 3.

Reviewer #1:
1. The tenses should to be corrected on line 1 of P9.

**Response:** Thank you for your comments from the reviewer. I have revised the original text and marked it in red. The content is as follows:

Based on the GEO, TCGA and GTEx databases, we reveal the possible molecular mechanism of the variable shear factor QKI in epithelial mesenchymal transformation (EMT) of oesophageal cancer.

2. The sentence should describe more accurately on line 3 of P9.

**Response:** Thank you for your comments from the reviewer. I have revised the original text and marked it in red.
3. The period should be in English format on line 2 of P11.

**Response:** Thank you for your comments from the reviewer. I have revised the original text and marked it in red.
4. Figure 1A has no legend; Figure 2A should provide a clear image; Figure 2D, 3B, 4A-4B should be more standardized; Each image in Figure 1-4 should provide a clear border, and you can refer to other papers in this journal.

**Response:** Thank you for your comments from the reviewer. The figure has been modified.
5. The table format should refer to other papers in this journal.

**Response:** Thank you for your comments from the reviewer. The table has been modified.
6. References should be in a uniform format, for example, reference 10 does not write the volume issue of the journal; reference 29 writes the month and day.

Response: Thank you for your comments from the reviewer. I have revised the original text and marked it in red.

Reviewer #2:
1- Authors mentioned the FPKM data was included in analyses, however for all the plots TPM was mentioned. Is it FPKM or TPM? I assume the logTPM (or FPKM) plus a constant was applied? Please clarify this part and fix all the axis or captions for figures (if needed).

Response: We are sorry for this mistake. In fact, the data format used in the differential analysis part of this study is TPM, and we have unified the descriptions between the figure and methods.

As you said, the data were first converted to log 2(TPM+1) and then subjected to differential analysis, which is also a common method used for analysis of the TPM data. We also referred to the GEPIA2 database (http://gepia2.cancer-pku.cn/#analysis) and adopt this way of drawing.
a. The differential expression analyses at Limma package are for counts data or log transformed expression data (FPKM or TPM).

Response: In this study, the data were first converted to log 2(TPM+1) and then subjected to differential analysis.
b. If FPKM was considered in the analyses, why not to include TPM (both TCGA and GTEX) since it is a kind gene length and library size normalized data.

**Response:** In fact, the data format used in the differential analysis part of this study is TPM, and we have unified the descriptions between the figure and methods.
c. It is important to share number of features or genes in analyses and any pre-process steps including remove low expressed genes, etc.
**Response:** Thank you for your suggestion. We have added relevant details in the methods section “The ComBat R package was used to remove the batch effect of data collected from TCGA and GTEx databases, after which data were merged. Then the limma R package was applied to standardize the gene expression data, and analyse the differential expression of QKI in normal control tissue samples and tumour tissue samples from esophageal carcinoma patients. The non-parametric Wilcoxon rank sum test was used when normality assumption was not satisfied…. we selected the path with the most significant difference with the normalized enrichment score (NES) p value and false discovery rate (FDR) q value as the criteria and drew the enrichment plot. Larger |NES| values of each gene set indicate that the gene set exhibits a stronger enrichment signal between the high and low expression groups of QKI. If |NES| values are more than 0, the gene set exhibits higher activity in the high expression group of QKI; if less than 0, the gene set is more active in the low expression group of QKI.”.
2- To increase the number of normal control tissue samples, GTEX was considered in addition to the TCGA. Authors combined these two studies. How about the potential batch(s) between these two cohorts? Any assessment was considered before integration?
**Response:** Thank you for your suggestion. It is necessary to merge TCGA and GTEx after removing batch effects, and our data analysis also includes this step. But the method description may be too concise, resulting in some details being lost. We have improved the corresponding method sections and have worked hard to review them again. We have added relevant details in the Screening of differentially expressed mRNAs and circRNAs section “The ComBat R package was used to remove the batch effect of data collected from TCGA and GTEx databases, after which data were merged. Then the limma R package was applied to standardize the gene expression data, and analyse the differential expression of QKI in normal control tissue samples and tumour tissue samples from esophageal carcinoma patients. The non-parametric Wilcoxon rank sum test was used when normality assumption was not satisfied”.

3- At the method section, please include more details about all analyses. Authors usually mentioned the R packages or soft wares (e.g., Cytoscape), while it is important to share more details. As an example
a. Differential expression analysis was done by limma package, while it is important to share more details about the Bayesian approach or moderated t statistics (just briefly)
**Response:** Thank you for your suggestion. We have improved the corresponding method section and have worked hard to review it again. We have added relevant details in the Screening for differentially expressed mRNAs and circRNAs section “Then the limma R package was applied to standardize the gene expression data, and analyse the differential expression of QKI in normal control tissue samples and tumour tissue samples from esophageal carcinoma patients. The non-parametric Wilcoxon rank sum test was used when normality assumption was not satisfied”.
4- FDR or q-value or adjusted p value? Please keep consistency for these terms across manuscript and the figures.
a. Authors mentioned most of the significant results based on p-values (GSEA, DE analysis). Why not adjusted for multiple test?
**Response:** We have checked the text and made corresponding revision to keep consistency for these terms across manuscript and the figures. In addition, we only performed correction for differential analysis and GSEA results but not for the results of correlation analysis.
5- Authors mentioned the median cut-off for expression (Low vs High) at GSEA analysis. I’m wondering to know the binary expression (Low vs High) was applied for differential expression analysis? I couldn’t follow. If yes, please mentioned at DE analysis section.
**Response:** The specific process of GSEA in this study is as follows: first, samples were divided into a high-expression QKI group and a low-expression QKI group. Then, each gene set was subjected to statistical analysis using the GSEA method (this study refers to the HALLMARK dataset) and an enrichment score (ES) was calculated. ES represents expression difference degree of the gene set in high-expression and low-expression samples; for each gene set, a background distribution is generated by random sampling and repeated calculation, after which p-value and FDR are calculated to judge whether the gene set is significantly enriched in the high-expression and low-expression samples. In the section of Gene set enrichment analysis (GSEA), we have detailed “In this study, we analysed the TCGA-ESCA dataset based on the QKI median expression value to analyse the high and low expression groups of QKI and compared the enriched pathway differences between the two groups by referring to the HALLMARK dataset. Then, we selected the path with the most significant difference with the normalized enrichment score (NES) *p* value and false discovery rate (FDR) *q* value as the criteria and drew the enrichment plot. Larger |NES| values of each gene set indicate that the gene set exhibits a stronger enrichment signal between the high and low expression groups of QKI. If |NES| values are more than 0, the gene set shows higher activity in the high expression group of QKI; if less than 0, the gene set is more active in the low expression group of QKI.”.
6- To predict the circRNA-miRNA-mRNA binding relationship, how the cut-off -0.2 was selected?
**Response:** In the correlation analysis results, we observed that the |r| of most results is mainly distributed within 0.1-0.3, that is, weak correlation. It can also be seen from Table 1 that if the screening criterion was set to |r| > 0.3, only 4 coding genes were screened, which was not conducive to intersecting with another gene set. In general, when multiple screening conditions exist at the same time, we appropriately lowered the screening criteria of |r|, and finally determined |r| > 0.2 as the threshold.
7- Figure 1: TPM or FPKM? What does star sign represent in Figure 1A? Please add details on caption.
**Response:** In Figure 1, it is TPM. In addition, we have explained “**** p.adj < 0.05***” in the legend.
8- The upset Figure 2A is not clear. Please re-generate the figure and add details about 7 groups. The color bar on Heatmap (green to red) represents which statistic? Please clarify.
**Response:** We have modified this figure and have provided detailed information in the results text “As shown in Fig. 2A, there was a large amount of alternative splicing in esophageal carcinoma. The height of the columns in the figure indicates the number of genes that undergo a certain alternative splicing. The first three columns are the individual parts of exon skip (ES), alternate terminator (AT) and alternate promoter (AP), the fourth column indicates the common part of ES and AP, and the fifth column indicates the common part of ES, AT and AP, which fully showed that the proportion of ES, AT and AP in esophageal carcinoma was relatively high.”. In addition, in this figure legend, we have detailed “Heatmap for differentially expressed genes in the GSE189830 dataset (normal group, n = 3; tumour group, n = 3) (C). The expression values are represented in different colors; red indicates high expression, and green indicates low expression. Darker node colors indicate more extreme high or low expression of the respective gene.”.
9- Table 1 only include the p-value. Why not the multiple correction tests approach included?
**Response:** We only performed correction for differential analysis but not for the results of correlation analysis ***(Gao Z,Xu J,Zhang Z, et al. A Comprehensive Analysis of METTL1 to Immunity and Stemness in Pan-Cancer. Front Immunol. 2022;13:795240. doi:10.3389/fimmu.2022.795240; Zhan X,Cheng J,Huang Z, et al. Correlation Analysis of Histopathology and Proteogenomics Data for Breast Cancer. Mol Cell Proteomics. 2019;18 (8 suppl 1):S37-S51. doi:10.1074/mcp.RA118.001232)***. We guess it may be because in the data analysis based on databases, for example, TCGA, the data were standardized. may be because in the process of data analysis based on databases such as TCGA, the data will be standardized and analyzed with log2(value+1). We chose Spearman or Pearson for correlation analysis based on the results of data normality test, so the correction of *p* value may not be so important. According to your suggestion, we have checked relevant literature and found that the p-value can be corrected by Bonferroni correction or FDR in correlation analysis, and we will also pay attention to it in future research.
10- Figure 3A only include TCGA? Or TCGA-GTEX? Is it log10 p-value or adj p-value? Please keep consistency and more precise about the title and axis and caption for figures.
**Response:** In fact, Figure 3A includes TCGA and GTEx. We have re-plotted this figure. The abscissa in the figure represents logFC, and the ordinate represents the corrected *p* value. We have changed the label of the ordinate to “p.adj”. In addition, we have marked the points of up-regulation and down-regulation in the figure, as well as the specific number for better understanding.
11- For each scatter plot. Please add the line as well.
**Response:** Thank you for your suggestion. We have added the line for each scatter plot.

12- To have reproducible research, is it possible to share code (GitHub) and data?

**Response:** We are more than willing to share data and if necessary, we can provide all raw data, upload as an attachment, or upload to GitHub.
