# Supplementary material for: The possible molecular mechanism underlying the involvement of the variable shear factor QKI in the epithelial-mesenchymal transformation of oesophageal cancer
Source: PLoS One. 2023 Jul 10;18(7):e0288403. doi: 10.1371/journal.pone.0288403 (PMC10332600; doi:10.1371/journal.pone.0288403)
Supplement: Attachment — Submitted filename: PONE-D-23-05659R1.docx [file pone.0288403.s003.docx]

The manuscript “The possible molecular mechanism underlying the involvement of the variable shear factor QKI in the epithelial-mesenchymal transformation of oesophageal cancer” demonstrated the variable shear factor QKI regulates two key circRNAs, hsa_circ_0006646 and hsa_circ_0061395, and downstream related miRNAs could relieve the targeted inhibition of EMT-related genes (IL11, MFAP2, MMP10, MMP1) and promoted the occurrence and development of oesophageal cancer, providing a new theoretical basis for screening prognostic markers of oesophageal cancer patients.

Comment:

1.The tenses need to be unified, such as line 17 in the Abstract, “promotes” should be “promoted”.

2. Line 1 in the Methods, “database” should be “databases”, pay attention to the use of single and plural numbers.

3. Line 19 in the Methods, Gene set enrichment analysis should be Gene set enrichment analysis (GSEA).

4. Line 20 in the Methods, “an” should be deleted.

5. Line 34 in the Methods, epithelial mesenchymal transformation should be EMT, abbreviation should be used.

6. Line 12 in the Results, “Fig. 1.” in the figure title should be “Figure 1”.

7. Line 21 in the Results, full name of ES, AP and AT should be given when first appeared.

8. Line 38 in the Results, “was” should be “is”.

9. Line 44 in the Results, the blank between “3” and “B” should be deleted.

10. Line 56 in the Results, the beginning space should be deleted.

11. Line 71 in the Results, “gastric cancer” should be “oesophageal cancer”.

12. The references should be checked carefully according to the PUBMED. And the format of references should be uniform.
