# Supplementary material for: The possible molecular mechanism underlying the involvement of the variable shear factor QKI in the epithelial-mesenchymal transformation of oesophageal cancer
Source: PLoS One. 2023 Jul 10;18(7):e0288403. doi: 10.1371/journal.pone.0288403 (PMC10332600; doi:10.1371/journal.pone.0288403)
Supplement: Attachment — Submitted filename: Response to Reviewers.docx [file pone.0288403.s004.docx]

Reviewer #1:

Comment:
1. The tenses need to be unified, such as line 17 in the Abstract, “promotes” should be “promoted”.

**Response**: Thank you for your comments from the reviewer. I have revised the original text and marked it in red. The content is as follows:

We further identified the significantly upregulated circRNAs and their corresponding coding genes in oesophageal cancer, screened the EMT-related genes that were significantly positively correlated with QKI expression, predicted the circRNA-miRNA binding relationship through the circBank database, predicted the miRNA-mRNA binding relationship through the TargetScan database, and finally obtained the circRNA-miRNA-mRNA network through which QKI promoted the EMT process.
2. Line 1 in the Methods, “database” should be “databases”, pay attention to the use of single and plural numbers.

**Response**: Thank you for your comments from the reviewer. I have revised the original text and marked it in red. The content is as follows:

The TCGA databases (https://www.cancer.gov) was used to download the gene expression data of normal control and oesophageal cancer patients, and the downloaded data format was transcripts per million (TPM) reads.
3. Line 19 in the Methods, Gene set enrichment analysis should be Gene set enrichment analysis (GSEA).

**Response**: Thank you for your comments from the reviewer. I have revised the original text and marked it in red. The content is as follows:

Gene set enrichment analysis (GSEA) is a computational method for determining whether a priori-defined gene set shows statistically significant consistent differences between two biological states.
4. Line 20 in the Methods, “an” should be deleted.

**Response**: Thank you for your comments from the reviewer. I have made modifications in the original text.
5. Line 34 in the Methods, epithelial mesenchymal transformation should be EMT, abbreviation should be used.

**Response**: Thank you for your comments from the reviewer. I have revised the original text and marked it in red. The content is as follows:

**Obtaining EMT-related genes from the MSigDB database**
6. Line 12 in the Results, “Fig. 1.” in the figure title should be “Figure 1”.

**Response**: Thank you for your comments from the reviewer. I have revised the original text and marked it in red. The content is as follows:

**Figure 1 Analysis of differential expression of QKI and pathway enrichment.**
7. Line 21 in the Results, full name of ES, AP and AT should be given when first appeared.

**Response**: Thank you for your comments from the reviewer. I have revised the original text and marked it in red. The content is as follows:

The first three columns are the individual parts of exon skip (ES), alternate terminator (AT), and alternate promoter (AP).
8. Line 38 in the Results, “was” should be “is”.

**Response**: Thank you for your comments from the reviewer. I have revised the original text and marked it in red. The content is as follows:

**QKI expression is significantly positively correlated with the expression of EMT-related genes (IL11, MFAP2, MMP10, MMP1)**
9. Line 44 in the Results, the blank between “3” and “B” should be deleted.

**Response**: Thank you for your comments from the reviewer. I have made modifications in the original text.
10. Line 56 in the Results, the beginning space should be deleted.

**Response**: Thank you for your comments from the reviewer. I have made modifications in the original text.
11. Line 71 in the Results, “gastric cancer” should be “oesophageal cancer”.

**Response**: Thank you for your comments from the reviewer. I have revised the original text and marked it in red. The content is as follows:

**Figure 4 Correlation analysis between EMT-related genes and survival of oesophageal cancer patients and QKI expression.**
12. The references should be checked carefully according to the PUBMED. And the format of references should be uniform.

**Response**: Thank you for your comments from the reviewer. I have revised the original text and marked it in red.

Reviewer #2: Authors addressed most of the comments, however two important comments as follows haven't seen any appropriate response:
1- For multiple test correlation analyses, no correction to control FDR or FWER. Why postpone to the future work?

**Response**: Thank you for your suggestion. We also strongly agree with the necessity of multiple tests. Therefore, we have added the following code to the correlation analysis. However, we have found that after multiple corrections, the correlation analysis results have not changed. The screenshot of the correlation analysis results before and after correction is shown below. The correlation analysis results can be found in the attached document, which was revised back together. The results before correction were "corResult before. txt", and the results after correction were "corResult after. txt". We have also modified table1 by replacing the original p value with the corresponding result of *p*. adjusted. Finally, we considered the reason why the correlation analysis results before and after correction did not change. Comparing the *p*-values before and after correction, the difference between p value and *p*. adjusted is relatively small, and we also use the r value as the screening criterion, indicating that the r value may have a greater impact on the screening results. Thank you again for your suggestion. We will also pay more attention to the rigor of bioinformatics analysis.

2- No GitHub link was shared. While, it is important to share codes to publish research reproducible (specially when the data are public and no limitation to get access).

**Response**: Thank you for your suggestion. My original data GitHub link is https://github.com/06cys2023/plos-one.
